# Effect of intralesional platelet-rich plasma (PRP) treatment on clinical and ultrasonographic parameters in equine naturally occurring superficial digital flexor tendinopathies – a randomized prospective controlled clinical trial

**DOI:** 10.1186/s12917-016-0826-1

**Published:** 2016-09-07

**Authors:** Florian Geburek, Moritz Gaus, Hans T. M. van Schie, Karl Rohn, Peter M. Stadler

**Affiliations:** 1 Clinic for Horses, University of Veterinary Medicine Hannover, Foundation, Bünteweg 9, 30559 Hannover, Germany; 2 Department of Equine Sciences, Faculty of Veterinary Medicine, Utrecht University, Yalelaan 112, 3584 CM Utrecht, Netherlands; 3 Institute for Biometry, Epidemiology and Information Processing, University of Veterinary Medicine Hannover, Foundation, Bünteweg 2, 30559 Hannover, Germany

**Keywords:** Horse, Tendon, Lameness, Ultrasonography, B-mode, Ultrasound tissue characterization, UTC, Platelet-rich plasma, PRP

## Abstract

**Background:**

Regenerative and anti-inflammatory effects on tendinopathies have been attributed to blood-derived biologicals. To date the evidence for the efficacy of autologous platelet-rich plasma (PRP) treatment of naturally occurring equine tendinopathies is limited. The purpose of this placebo-controlled clinical trial was to describe the effect of a single treatment of equine superficial digital flexor tendon (SDFT) disease with PRP on clinical and ultrasonographic parameters. Twenty horses with naturally occurring tendinopathies of forelimb SDFTs were randomly assigned to the PRP-treated group (*n* = 10) or control group (*n* = 10) after clinical and ultrasonographic examination. The SDFTs received an intralesional treatment with autologous PRP or were injected with saline, respectively (day 0). All horses participated in a standardized exercise programme and were re-examined clinically, with B-mode ultrasonography (5 times at regular intervals) and ultrasound tissue characterization (week 12 and 24 after treatment) until week 24. Long-term performance was estimated via telephone inquiry.

**Results:**

Compared to day 0, lameness decreased significantly by week 8 after treatment with PRP and by week 12 in the control group. Ultrasonographically there was no difference in the summarized cross sectional area between the groups at any time point. Ultrasound tissue characterization showed that echo types representing disorganized matrix decreased significantly throughout the observation period in the PRP-treated group. Echo type II, representing discontinuous fascicles, not yet aligned into lines of stress was significantly higher 24 weeks after PRP treatment. Eighty percent of the PRP treated horses reached their previous or a higher level of performance after 12 months compared to 50 % in the CG. After 24 months these proportions were 60 % and 50 %, respectively.

**Conclusions:**

A single intralesional treatment with PRP up to 8 weeks after onset of clinical signs of tendinopathy contributes to an earlier reduction of lameness compared to saline treatment and to an advanced organization of repair tissue as the fibrillar matrix is getting organized into fascicles while remodelling continues. Long term, PRP treatment has the potential to increase the number of horses reaching their previous level of performance. Earlier treatment of tendinopathy with PRP should be considered to enhance these effects.

## Background

Tendinopathy of the superficial digital flexor tendon (SDFT) is a common injury in Thoroughbred racehorses and other horse breeds [1, 2] and is regarded as a career-limiting disease [3]. Clinical injury is mostly strain induced and characterized by chronic degeneration after repetitive microtrauma in sport horses [3]. Tendinopathy may also be caused by a single external percutaneous trauma, such as a kick [4]. A plethora of substrates for intralesional injection with a potentially regenerative effect on tendinopathy are currently under investigation [5, 6]. Among these are platelet concentrates such as platelet-rich plasma (PRP) [7].

Concentrated platelets release numerous cytokines and growth factors, e.g. platelet derived growth factor-BB (PDGF-BB), transforming growth factor-ß (TGF-ß) and vascular endothelial growth factor. These factors are known to exercise specific actions during tendon healing (for review see Docheva et al. 2015) [6, 8]. PDGF-BB, e.g. stimulates tendon healing depending on its concentration [9]. PRP has been shown to enhance tenocyte proliferation [10], collagen and matrix synthesis [11, 12] and to influence vascular density [13].

PRP has shown significant enhancing effects on tendon healing such as improved biomechanical properties in a surgical model of equine SDFT lesions [13, 14] and is increasingly used for intralesional treatment of tendinopathy and desmopathy in humans [15] and horses [16–19] in clinical practice. However, the efficacy of PRP treatment on tendinopathy is seen controversially in human studies: In a randomized controlled clinical trial on chronic human Achilles tendinopathy intralesional PRP treatment showed no beneficial effects compared to saline injection [20]. These results are supported by a further randomized controlled study demonstrating no differences in elasticity modulus of PRP- versus non-treated Achilles tendons [21]. In other clinical studies without control groups PRP treatment of chronic Achilles and patellar tendinopathy significantly improved symptoms and function [22, 23]. To the best of our knowledge the effect of a single intralesional treatment with autologous PRP on equine naturally occurring SDF tendinopathy has not been documented in a prospective randomized placebo-controlled clinical study with long term follow-up in the literature to date (for review see Brossi et al. 2015) [18].

Autologous platelet concentrates are prepared by centrifugation or by gravitational cellular filtration [24]. Blood samples from different horses and the use of different non-standardised and commercial kits for the preparation of PRP lead to differences in the platelet, leucocyte and growth factor content [24]. Different breed, age and gender influences growth factor and cytokine release of platelets and leucocytes [25, 26]. The optimal platelet and leucocyte contents in PRP are a point of debate. Recent investigations have shown that a moderate increase in platelets seems to enhance equine tenocyte growth and collagen synthesis more effective than high platelet concentrations [27, 28]. High leucocyte counts in PRP were observed to enhance the inflammatory reaction in rabbit tendons [29].

The process of tendon healing is mainly divided into three phases which merge into each other: The acute inflammatory phase (<10−14 days) is characterized by phagocytosis of disrupted tendon tissue and demarcation of injured tendon tissue. A fibroproliferative callus is formed during the proliferative phase (4–45 days), while collagen fibrils are organised into tendon bundles during the phase remodelling (45–120 days; < 3 months) which may be divided into an consolidation (earlier) and maturation (later) phase [3, 30].

A critical parameter to determine the success of tendinopathy treatment is functionality, which can be characterized by lameness evaluation, long term recurrence rate [2, 31] and biomechanical testing [32]. To determine the quality of repair tissue tendon needle biopsies have been used successfully [33]. Ultrasound based modalities such as B-mode ultrasonography [34] and colour Doppler ultrasonography [13] have been established to monitor the process of tendon healing non-invasively. However, B-mode ultrasonography has several limitations, e.g. lack of axial information, operator-dependence, influence of ultrasound beam angle and limited resolution that impair its sensitivity [35, 36]. Ultrasound tissue characterization (UTC) is a new technique to quantify tendon integrity based on a computerized analysis of the stability of echo-patterns in contiguous US images [37, 38]. Based on the echo-pattern stability, 4 different echo types can be discriminated, with histo-morphology of tendon specimen as reference test [37, 39]. Ultrasound tissue characterization has been shown a viable diagnostic tool to monitor experimental tendinopathy in horses [39–42] and is increasingly used for the quantification of Achilles and patellar tendon integrity in humans [43, 44].

### Aim of current study

The aim of the present study was to support the hypothesis that a single intralesional injection of autologous PRP into SDFT lesions up to 8 weeks after clinical onset (1) has a clinically detectable effect and (2) leads to improved ultrasonographic parameters (3) influences long term functionality.

## Methods

Inclusion criteria for client-owned adult horses was an anamnesis of clinical signs of uni- or bilateral SDFT disease in front limbs for up to 8 weeks (56 days) prior to the presentation at the Equine Clinic of the University of Veterinary Medicine, Hannover, Foundation, or to collaborating veterinarians. Horses were only included if the clients agreed to the study design and tendons had not received intralesional injections before. Twenty horses with a mean age of 8.46 years (range: 4–21 years) and a mean bodyweight of 548 kg (range: 398–644 kg) met the inclusion criteria (Table 1). Horses were randomly assigned to the group treated with PRP (PRPG, *n* = 10) or the control group (CG, *n* = 10). The PRPG comprised 7 Warmbloods (70 %), 1 Haflinger (10 %), 1 New Forest Pony (10 %) and one Pinto (10 %). Eight Warmbloods (80 %), 1 Andalusian (10 %) and 1 German Riding Pony (10 %) were included in the CG (Table 1). The study was carried out between 2012 and 2014.

**Table 1:** Signalement, clinical history, diagnostic data and treatment of 20 horses with SDFT lesions

Horse number	Breed	Age (y)	Gender	Purpose used for	Reported duration of SDFT tendinopathy until initial exam (w)	Reported initiating event	Limb affected	Maximal injury zone (MIZ)	Lesion type
PRP-group									
1	Hannoverian	4	G	Dressage	2	Unknown	LF	2a, lat	Marginal
2	Hannoverian	8	M	Dressage	1	Training	LF	3a, palm	Marginal
3	Haflinger	21	G	Trained horse	2	Pasture turnout	LF	2b	Diffuse
4	Trakehner	5	S	Dressage	4	Pasture turnout	RF	3b, med	Marginal
5	Oldenburger	16	G	Police horse	2	Ride	RF	2b	Diffuse
6	Rhenish Warmblood	13	M	Dressage	2	Unknown	RF	3a	Core
7	Hannoverian	6	M	Dressage	2	Running free	LF	2b, palm	Marginal
8	New Forest Pony	13	G	Pleasure	3	Pasture turnout	LF	2b	Diffuse
9	Hannoverian	11	G	Eventing	8	Training	RF	3a	Core
10	Pinto	20	G	Pleasure	7	Unknown	LF	2b, lat	Marginal
		∅ 11.7							
Control group									
1	Oldenburger	9	M	Jumping	4	Jumping	LF	3a, lat	Marginal
2	Hannoverian	5	G	Dressage	6	Unknown	LF	3a, med	Marginal
3	Andalusian	18	M	Pleasure	3	Pasture turnout	LF	2b	Diffuse
4	Hannoverian	15	M	Dressage, jumping	2	Training	LF	2b, med	Marginal
5	German Riding Pony	9	M	Jumping	1	Jumping	LF	3a, lat	Marginal
6	Hannoverian	12	G	Police horse	2	Running free	RF	1b, lat	Marginal
7	Hannoverian	20	M	Pleasure	2	Unknown	RF	2a	diffuse
8	Rhenish Warmblood	7	G	Eventing	2	Training	LF	3a	Core
9	Hannoverian	10	G	Eventing	6	Jumping	LF	2b, lat	Marginal
10	Hannoverian	6	G	Pleasure	4	Unknown	RF	3a	Diffuse
		∅ 11.1							

*SDFT* superficial digital fexor tendon, *w* week(s), *y* years, *S* stallion, *M* mare, *G* gelding, *LF* left front, *RF* right front, *PRP-group* SDFT treated with intralesional injection of platelet-rich plasma, *Control group* SDFT treated with intralesional saline injection, *lat* lateral, *med* medial, *palm* palmar, ∅ mean

### Clinical examination

All horses were examined clinically on the day of first presentation (day 0). This examination included assessment of lameness (5 grade score) [45] and palpable signs of inflammation scored semi-quantitatively by palpation (skin surface temperature in the palmar metacarpal region, sensitivity of the SDFT to palpation: 0 = no abnormality, 1 = mild abnormality, 2 = moderate abnormality, and 3 = severe abnormality) [46]. All clinical examinations throughout the study were performed by the same person (M.G.).

### B-mode ultrasonography

Horses were sedated with romifidine (0.04–0.08 mg/kg intravenously [IV]) and butorphanol (0.01 mg/kg [IV]). Injured and contralateral SDFTs were examined by one examiner with B-mode ultrasound on day 0 in a transverse and longitudinal fashion with a linear 6–15 MHz linear scanner (Logiq E9, GE Healthcare, Wauwatosa, WI, USA) using a standoff (GE 12 L 2302652 Standoff, Veterinary Sales & Service Inc., Stuart, USA), according to the seven zone designations as previously described [47, 48]. Settings were 13 MHz, gain 52, depth 25 mm, focus position 15 mm. Images were stored digitally and analysed retrospectively with a DICOM workstation program (easyVET®, IFS Informationssysteme, Hannover, Germany) by one observer (M.G.) being unaware of the treatment modality. The following parameters were evaluated to determine the degree- and time-related changes of the lesions: maximal injury zone (MIZ), summarized cross sectional areas of tendon (total cross-sectional area, T-CSA). Echogenicity and fibre alignment were graded semiquantitatively at each zone and the scores for all levels were summarized (total echo score = TES, total fibre alignment score = T-FAS). Echogenicity was assigned to 0 (normoechoic), 1 (hypoechoic), 2 (mixed echogenicity), and 3 (anechoic), and fibre alignment was graded according to the estimated percentage of parallel fibres in the lesion: 0 (>75 %), 1 (50–74 %), 2 (25–49 %), and 3 (<25 %) [34, 48].

### Ultrasound tissue characterization (UTC)

Injured SDFTs were scanned with the commercially available ultrasound tissue characterization (UTC) unit (UTC scan unit, configuration 2011, UTC Imaging B.V., 6171 GD Stein, Netherlands) on day 0. It consisted of 5–10 MHz linear transducer (LA T Transducer, Terason Ultrasound, Teratech Corporation, Burlington, USA) which was fixed in transverse position, perpendicular to the integrated stand-off, in a motorized tracking device (UTC scan unit, configuration 2011, Fa. UTC Imaging B.V., GD Stein, Netherlands) that moves the transducer automatically along the tendon. Within the UTC protocol the instrumental settings for the SDFT were selected which facilitated standard settings like persistence, depth, focal point, gain, TGC-curve, to be used for all scans. During scanning, transverse US images were captured at regular distances of 0.2 mm over a distance of 12 cm and stored real-time in a high-capacity laptop computer (MacBook Pro® 17“, Apple, Cupertino, USA). Compilation of these images created a 3-D block of US information that was used for quantification of stability of contiguous echo-patterns. Based on the echo-pattern stability, 4 different echo-types were discriminated, with histo-morphology of tendon specimen as reference test: type I, generated by intact and fully aligned fascicles (secondary collagen bundles); type II, generated by discontinuous, waving, not (yet) aligned and/or swollen fascicles (secondary collagen bundles); type III, generated by a mainly fibrillar matrix (collagen fibrils not/no yet organized into fascicles) and type IV, generated by a mainly amorphous matrix and/or fluid [37, 39].

Scanning for UTC was performed in standing, fully sedated horses while all limbs were weightbearing. As the UTC device allows to scan a metacarpal segment of approximately 12 cm, the device was applied to the metacarpal area in a way during the initial exam that the zone of maximal injury (MIZ) was centered in the middle of the standoff. The distance from the most prominent point of the accessory carpal bone to the proximally located edge of the standoff was measured and recorded to ensure that the same segment would be scanned during following UTC scans. Additionally this point was marked in the horse’s latero-palmar metacarpal haircoat with a shaving blade. A corresponding segment of the SDFT of the opposite front limb was scanned in the same manner.

With the help of the software, the stability of the echo pattern of corresponding pixels over 9 contiguous transverse images was analysed by UTC algorithms. Images were controlled for movement artifacts and repeated if necessary. Data sets were stored digitally on the computer until final analysis.

A single scan of each tendon was analyzed retrospectively with the analyzing-software (UTC2011® Analyser V1.0.1, Fa. UTC Imaging, 6171 GD Stein, The Netherlands) by one observer (M.G.) being blinded to the treatment modality: A 6 cm long tendon segment was defined from 3 cm distally and proximally to the MIZ, respectively, on the colour coded longitudinal image produced during the first examination (day 0). Within this segment, the entire cross sectional area of the SDFT was analyzed using every 15^th^ consecutive transverse image, i.e. every 3 mm. Ratios of echo types were analyzed quantitatively as fractions of the entire cross sectional area and mean values for the proportion of each type were calculated. The distance from the center of MIZ to the proximal end of the scan was measured to retrieve the MIZ and thereby the segment to be analyzed in control scans during the remaining examination period.

### Preparation of PRP, Intralesional treatment, controlled exercise and follow-up examinations

Fiftyfour millilitres of autologous blood were collected by a single venipuncture of one jugular vein into a 60 ml syringe preloaded with 6 ml of the anticoagulant acid citrate dextrose (ACD-A). Approximately 5 ml of whole blood was aspirated into a tube containing EDTA (Vacuette®, Greiner Bio-One International AG, Kremsmünster, Austria) for later cell counts. To ensure that the whole blood and anticoagulant homogenously mix, the syringe was inverted gently 4 times. The content was transferred aseptically into bag A of a commercially available PRP preparation system (Osteokine®, Orthogen, Düsseldorf, Germany). The bag system was inserted in a swingout bucket of a centrifuge (Universal 300®, Andreas Hettich GmbH & Co. KG, Tuttlingen, Germany) and a corresponding swingout bucket was filled with a counterweight. According to the manufacturer’s instruction the first centrifugation step was performed at a relative centrifugation force of 900 g for 3 min. The plasma including the buffy coat was transferred via the connecting tube to bag B by gently rolling the bottom of bag A upwards with a clamp. After closing the ports between bag A and B the system was centrifugated for 10 min at a relative centrifugation force of 1470 g. The supernatant platelet poor plasma (PPP) was aspirated via the withdrawal port. The remaining pellet of platelets and residual erythrocytes was resuspended by reinjecting a volume of 3 ml of PPP into bag B. The resulting PRP was completely aspirated from bag B which led to a volume of 4 ml. An aliquot of 1 ml PRP was transferred to a tube containing EDTA (Vacuette®, Greiner Bio-One International AG, Kremsmünster, Austria), while the remaing 3 ml were used for intralesional injection. Platelet- and leucocyte content was determined from whole blood and PRP with an automated cell counter (KX-21 N®, Sysmex, Norderstedt, Germany).

Horses were sedated for the intralesional injections with detomidine hydrochlorid (0.01–0.03 mg/kg [IV]) and butorphanol (0.04–0.05 mg/kg [IV]), and the medial and lateral palmar nerves were anaesthetized 2 cm distal to the carpometacarpal joints with 2,5 ml of a 2 % mepivacain solution, respectively.

After aseptic preparation of the skin, superficial digital tendon lesions were injected under sonographic guidance from the lateral aspect of the tendon perpendicularly to its long axis directly into the most hypoechoic areas through a 22G (∅ 0.7 mm, 30 mm) canula (BD Microlance^TM^ 3, Becton Dickinson S.A., Fraga, Huesca, Spain) while the limb was weight-bearing. SDFTs allocated to the PRPG received an intralesional treatment of 3 ml into the tendon defect (day 0). Tendons in the CG were injected intralesionally with 3 ml 0,9 % sterile saline solution (Isotone Kochsalzlösung 0,9 %®, B. Braun Melsungen, Melsungen, Germany). The volume of PRP or saline was equally distributed to 3 sites. The first injection was performed at the maximal injury zone. Further injection sites were 1–2 cm distally and proximally to the first injection. A padded distal limb bandage was applied and left in place during a period of 3 days box rest in all horses. The contralateral SDFT was not injected. All front hooves of all horses were shod with a closed horse shoe with a straight bar [49]. All horses participated in a gradually increasing exercise programme adapted from Bosch et al. [14]. Depending on the anamnestically reported duration of clinical SDF tendinopathy of less than 4 weeks or 5–8 weeks, horses were either given box rest for another 4 weeks or commenced with handwalking for 10 min, respectively (Table 2). The horses were re-examined clinically and with B-mode ultrasonography at 4, 8, 12, 18 and 24 weeks after injection. Ultrasound tissue characterization was repeated 12 and 24 weeks after treatment.

**Table 2:** Gradually increasing exercise programme adapted from Bosch et al. [14] and modified

Exercise/day	Period
Box rest	4 weeks
10 min walk	Weeks 5–8
20 min walk + 2 × 3 min trot	Weeks 9–12
30 min walk + 15 min trot	Weeks 13–15
30 min walk + 15 min trot + 3 × 1 min gallop	Weeks 16–18
35 min walk + 15 min trot + 3 × 5 min gallop	Weeks 19–24

*min* minutes

All owners were asked about the performance of their horses via telephone inquiry 12 and 24 months after treatment according to the following categories: reached previous or higher level of performance; was ridden at a lower level of performance; was retired; died.

### Statistical analysis

Analysis of data was performed using SAS® Version 9.3 (SAS Institute, Cary, NC, USA). The level of significance was set at *p* < 0.05. All values in the graphs are expressed as arithmetic mean values with standard error ($\overline{\mathrm{X}}$ ± SEM). The assumption of normality was tested using the Kolmogorov-Smirnov test and visual assessment of qq-plots of model residuals. In the case of rejection of normal distribution, distribution-free nonparametric methods were applied. Fisher’s exact test was applied by evaluation of contingency tables to test the differences between groups (PRP-treated group and control group) on each examination day with regard to the parameters degree of lameness, sensitivity to palpation and skin surface temperature. To work out the differences of these parameters within a group between examination days, non-adjusted p-values were used provided that the global test was significant (*p* < 0,05) or showed a tendency to significance (*p* = 0,05 - 1). The influence of the groups and time points on the ultrasonongraphic parameters (T-CSA, T-FAS and TES) was tested using a two-way analysis of variance for independent samples (groups) and repeated measurements (time points), followed by the Tukey *post hoc* test for multiple pairwise comparisons. P-values were used in terms of comparison to assess differences of parameters between individual days of examination within a group and *vice versa*, and to determine the differences between the two groups on the respective examination day.

## Results

### Clinical examination

#### Lameness

**Fig. 1:**
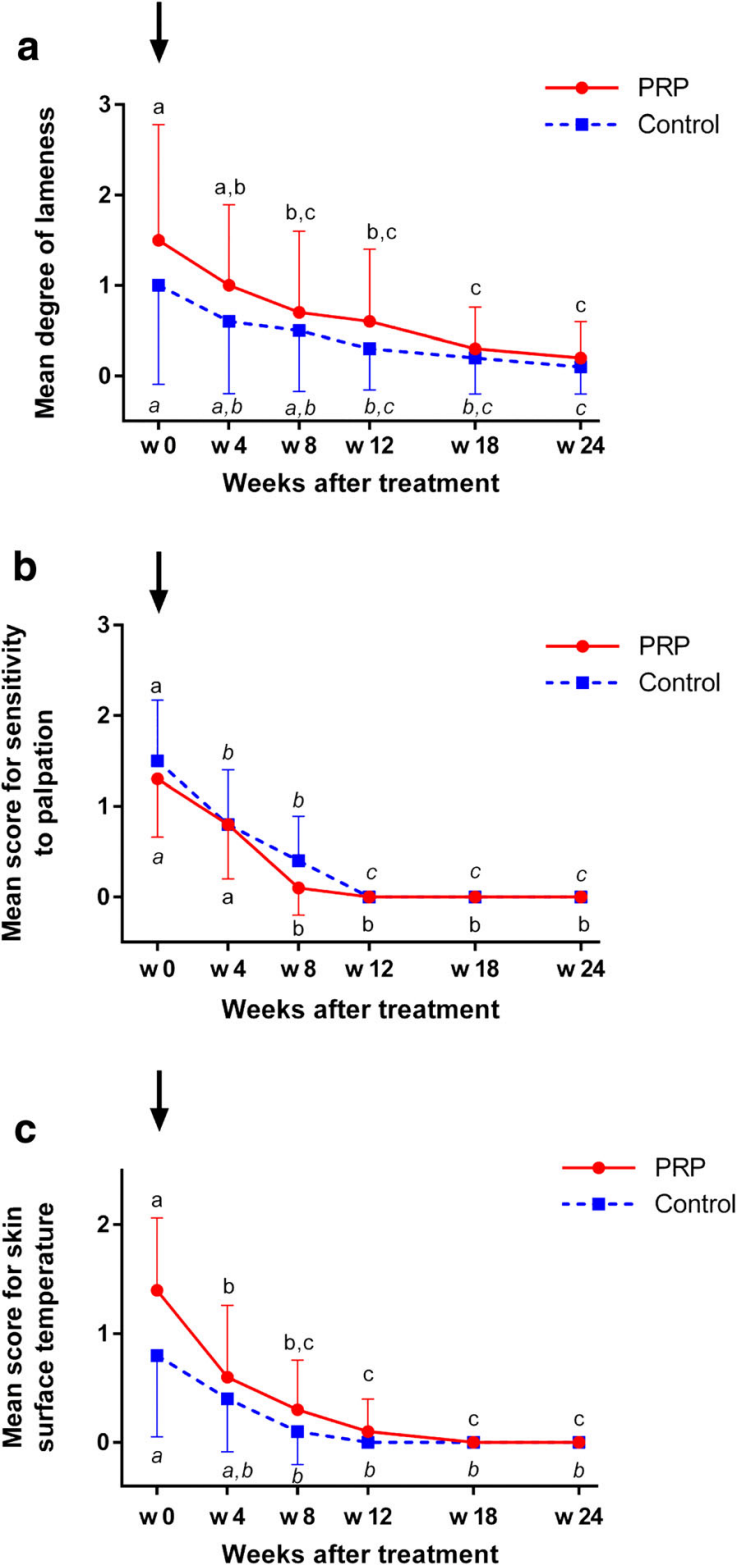
Clinical findings after PRP and saline treatment of SDFT lesions over time. **a** Degree of lameness. **b** Scores for sensitivity to palpation. **c** Scores for skin surface temperature in the palmar metacarpal region. Mean ± SE; global test for PRPG and CG *p* < 0.001; Different letters (PRP normal and control italic) indicate significant differences (*p* < 0.05) within groups; PRPG = PRP-group: *n* = 10 limbs, SDFTs treated intralesionally with PRP; CG = control group: *n* = 10 limbs, SDFTs treated intralesionally with saline; black arrow = day 0: diagnosis, intralesional injection of PRP/saline; PRP = platelet-rich plasma; w = weeks; SDFT = superficial digital flexor tendon

On day 0, 60 % (*n* = 6) of the horses included in the PRPG were lame. Fifty percent (*n* = 5) of the control horses showed a lameness. In all horses it was the lame limb where tendinopathy was diagnosed. After 24 weeks 2 horses in the PRPG were still lame and 1 horse in the CG was lame. The mean degree of lameness did not differ between groups on any day of examination. Compared to day 0 lameness decreased significantly by week 8 after treatment within the PRPG (*p* = 0.011) and it decreased significantly by week 12 in the CG (*p* = 0.014) (Fig. 1a).

### Clinical signs of inflammation

No statistically significant differences between the two groups with regard to scores for sensitivity to palpation and skin surface temperature were observed on day 0 and during the entire observation period (Fig. 1b, c). Within the PRPG scores for sensitivity to palpation of the SDFTs decreased significantly for the first time between week 4 and week 8 (*p* = 0.003) and in the CG between day 0 and week 4 (*p* = 0.02). From week 12 until the end of the observation period no painful reaction could be elicited in all horses (Fig. 1b). Scores for skin surface temperature (Fig. 1c) decreased significantly between day 0 and week 4 (*p* = 0.001) in the PRPG. Within the CG it took until week 8 that skin surface temperature decreased significantly (*p* = 0.001). From week 8 onwards scores for this parameter remained at a low level up to 24 weeks in both groups.

### B-mode ultrasonography

**Fig. 2:**
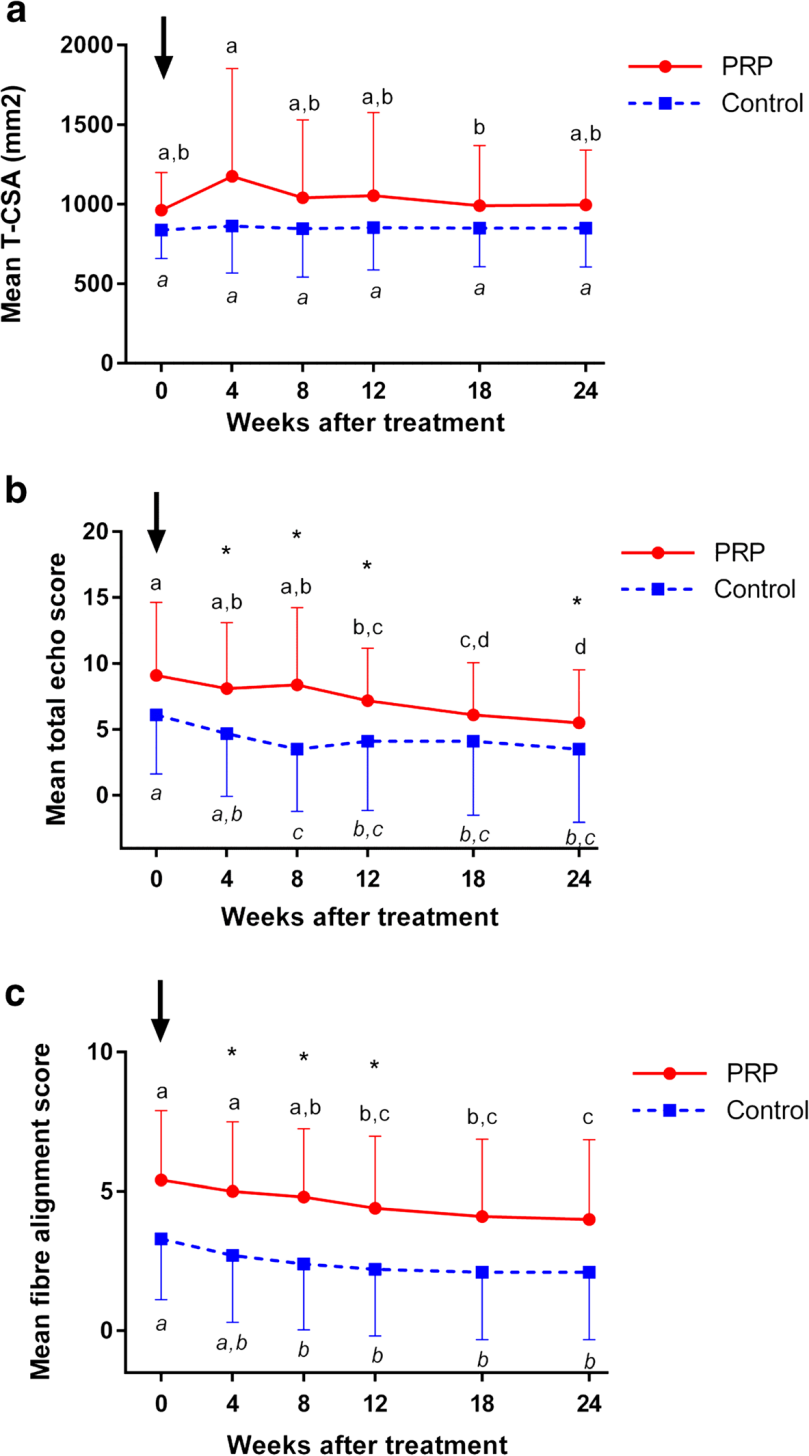
B-mode ultrasonographic measurements. **a** Total cross sectional area of PRP- and saline treated SDFTs over time (global test: PRPG *p* = 0.079, CG *p* = 0.999). **b** Total echo scores of PRP- and saline treated SDFT lesions over time (global test: PRPG and CG *p* < 0.001). **c** Fiber alignment scores of PRP- and saline treated SDFT lesions over time (global test: PRPG and CG *p* < 0.001). Mean ± SE; Values labelled with asterisk (*) differ significantly (*p* < 0.05) between groups. Different letters (PRP normal and control italic) indicate significant differences (*p* < 0.05) within groups; PRPG = PRP-group: *n* = 10 limbs, SDFTs treated intralesionally with PRP; CG = control group: *n* = 10 limbs, SDFTs treated intralesionally with saline; black arrow = day 0: diagnosis, intralesional injection of PRP/saline; PRP = platelet-rich plasma; FAS = fiber alignment score; SDFT = superficial digital flexor tendon; TCSA = total cross sectional area; TES = total echo score; w = weeks

The horses included were presented with core lesions (3 horses), marginal lesions (11 limbs) or diffuse lesions (6 limbs) of the SDFT (Table 1). Control horses no. 3 and 7 had ultrasonographic signs of scarring of the SDFT in the contralateral non-lame limb on day 0. The MIZ of most lesions was located in zones 2b and 3a (80 % of horses in PRPG and CG, respectively), followed by zone 2a (10 % of horses in PRPG and CG, respectively), and zones 1b (10 % of horses in PRPG) and 3b (10 % of horses CG), respectively). There was no difference in T-CSA between the groups at any time point (Fig. 2a). Compared to the contralateral SDFT, T-CSA was significantly higher in PRP treated and in control limbs than in the respective contralateral limbs during the whole observation period (*p* < 0.05). Within the PRP group T-CSA was significantly higher (*p* = 0.024) only in week 4 as compared to week 18 (Fig. 2a). TES was significantly higher in the PRPG than in the CG at weeks 4, 8, 12 and 24 (*p* = 0.0397, *p* = 0.0069, *p* = 0.00297, *p* = 0.0424) (Fig. 2b); and T-FAS was significantly higher in the PRPG than in the CG at weeks 4, 8 and 12 (*p* = 0.0387, *p* = 0.0140, *p* = 0.0283) (Fig. 2c). Regarding the progression of TES and T-FAS they decreased significantly between day 0 and week 12 in the PRPG (TES: *p* = 0.002; T-FAS: *p* = 0.001) and between day 0 and week 8 the CG (TES: *p* < 0.001, T-FAS: *p* = 0.023) (Fig. 2b, c).

### Ultrasound tissue characterization

**Fig. 3:**
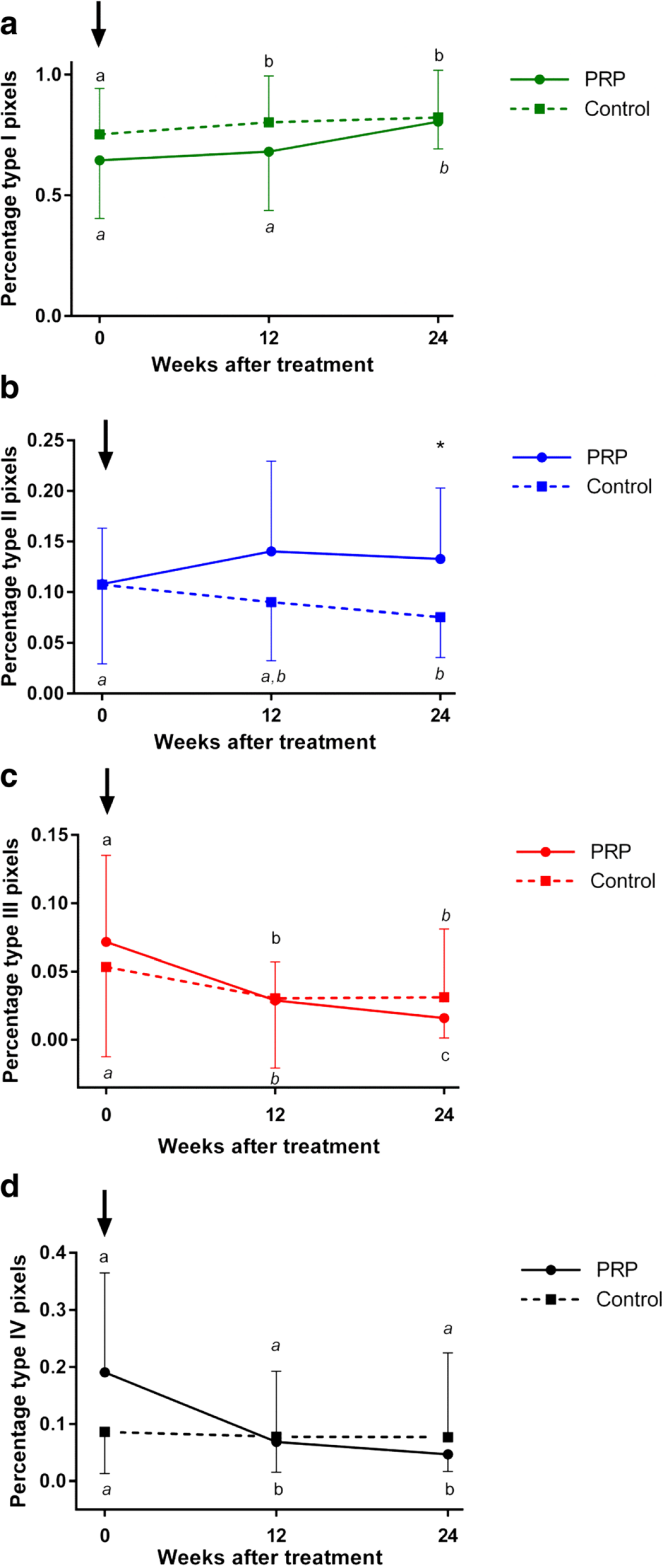
Ultrasound tissue characterization: Proportion of echo-types I-IV. Proportion of echo types **a** I, **b** II, **c** III and **d** IV for PRP and saline treated SDFTs; PRP-group: solid lines; Control group: dotted lines; Mean ± SE; Values labelled with asterisk (*) differ significantly (*p* < 0.05) between groups. Global tests: echo type I: PRPG *p* = 0.099, CG *p* = 0.004; echo type II: PRPG *p* = 0.204, CG *p* = 0.057; echo type III: PRPG p < 0.001, CG *p* = 0.002; echo type IV: PRPG *p* < 0.001, CG *p* = 0.940; Different letters (PRP normal and control italic) indicate significant differences (*p* < 0.05) within groups; PRPG = PRP-group: *n* = 10 limbs, SDFTs treated intralesionally with PRP; CG = control group: *n* = 10 limbs, SDFTs treated intralesionally with saline; PRP = platelet-rich plasma; SDFT = superficial digital flexor tendon; w = weeks

**Fig. 4:**
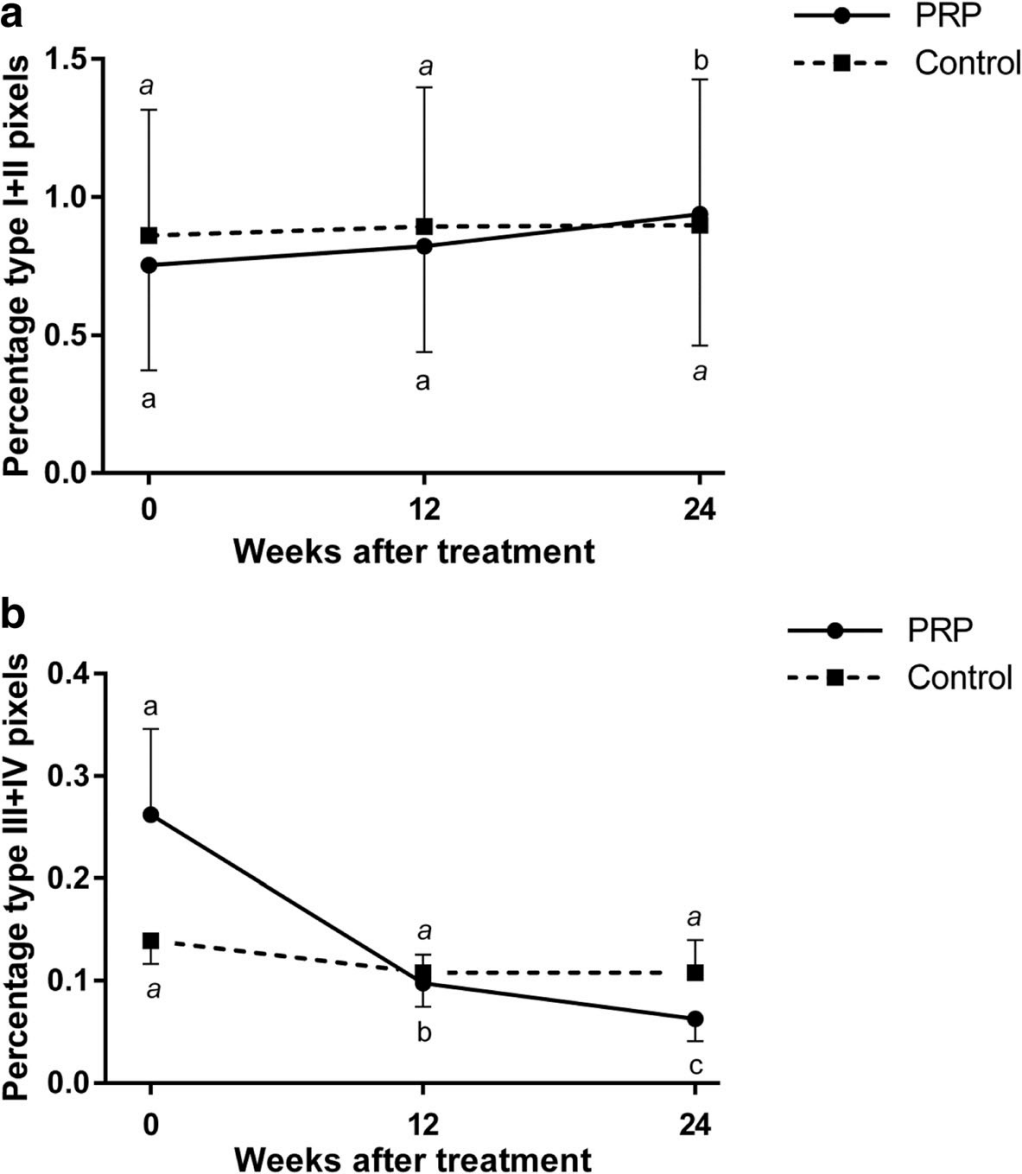
Ultrasound tissue characterization: Proportion of combined echo-types (**a**) I + II and (**b**) III + IV. PRP-group: solid lines; Control group: dotted lines; Mean ± SE; Global tests: combined echo type I + II: PRPG *p* = 0.0826, CG *p* = 0.318; combined echo type I + II: PRPG p < 0.001; CG *p* = 0.362; Different letters (PRP normal and control italic) indicate significant differences (*p* < 0.05) within groups; PRPG = PRP-group: *n* = 10 limbs, SDFTs treated intralesionally with PRP; CG = control group: *n* = 10 limbs, SDFTs treated intralesionally with saline; PRP = platelet-rich plasma; SDFT = superficial digital flexor tendon; w = weeks

The percentages of echo types I, III, IV (Fig. 3a, c, d), combined types I + II and combined types III + IV (Fig. 4) did not differ between groups at any time point. The percentage of echo type II was significantly higher (*p* = 0.0492) in the PRPG than in the CG at the end of the observation period (Fig. 3b).

In the PRPG percentages of combined type I + II pixels representing fascicular structures increased significantly between week 12 and 24 (*p* = 0.009) (Fig. 4a). Combined types III + IV percentages representing disorganized matrix decreased significantly between day 0 and week 12 (*p* < 0.001) and again between weeks 12 and 24 (*p* = 0.024) in the PRPG (Fig. 4b). In the CG both, combined type I + II and combined type III + IV did not change throughout the observation period (*p* > 0.05) (Fig. 4).

In the CG echo type I, i.e. intact and fully aligned fascicles increased significantly between day 0 and week 12 (*p* = 0.001) and after PRP treatment between week 12 and 24 (*p* = 0.012) (Fig. 3a). Echo type III representing mainly fibrillary matrix decreased significantly in both groups between day 0 and week 12 (*p* < 0.001). It further decreased significantly between week 12 and 24 in the PRPG (*p* = 0.006) while it remained on the same level in the CG (Fig. 3c). The percentage of echo type IV, i.e. amorphous matrix/fluid decreased siginificantly between day 0 and week 12 in the PRPG (*p* < 0.001). In the CG this type remained on the same level throughout the whole observation period (Fig. 3d).

### Concentration of platelets and leucocytes

The mean number of platelets was 157.3 ± 35.9 × 10^3^/μl in whole blood and 892.37 ± 364.7 × 10^3^/μl in PRP. Leucocyte content was 7.8 ± 1.5 × 10^3^/μl in whole blood and 14.1 ± 7.0 × 10^3^/μl in PRP. Compared to whole blood platelets and leucocytes were concentrated significantly by factor 5.67 (*p* = 0.0001) and 1.81 (*p* = 0.0139), respectively in PRP.

### Long-term follow-up

One horse allocated to the PRPG and one control horse developed recurrence of tendon injury in the treated limb within 2 years post diagnosis. One PRP treated horse developed tendinopathy in the SDFT of the contralateral front limb. Twelve months after treatment 80 % (8/10) of the horses allocated to the PRPG performed at the previous or at a higher level while this was the case in 50 % (5/10) of the control horses. Twenty-four months after treatment 60 % (6/10) were still ridden at the previous or higher level of performance in the PRPG while this was the case in 50 % (5/10) of the control horses. Horses that were ridden at a lower level of performance made up 10 % (1/10) of the PRP treated horses after 12 months and 20 % (2/10) after 24 months. In the CG 40 % (4/10) of the horses performed at a lower level after 12 months and 20 % (2/10) after 24 months. One horse included in the PRPG was retired within 12 months while one control horse was retired between 12 and 24 months after treatment. One PRP treated horse died of reasons unrelated to tendinopathy between 12 and 24 months and one control horse died within 12 months after treatment. Long term outcome did not differ significantly between groups.

## Discussion

Results of this controlled clinical trial demonstrate that, compared to controls, single intralesional PRP treatment leads to an earlier reduction of the degree of lameness until week 8 and to an earlier decrease in palpable skin temperature of the SDFT region until week 4 after treatment. At 24 weeks after PRP treatment ultrasound tissue characterization showed a significantly higher percentage of type II echoes which, in combination with decrease of type III and IV, is indicative for advanced repair as the fibrillar matrix is getting organized into fascicles, although not yet aligned into lines of stress. Eighty percent of the PRP treated horses reached their previous or a higher level of performance after 12 months and 60 % continued to perform at this level after 24 months compared to 50 % in the CG at both time points.

Because of the generally limited availability of adequate patients for a controlled experimental study in a clinical setting, horses of different ages, breeds and types of use were included in the current study. It is established that the potential for tendon healing is greater in younger horses [50] and that among others male gender and high body weight are risk factors for tendinopathy [51]. The CG contains more horses used for show jumping and eventing which generally implies a higher tendon load and a potentially higher risk for self-inflicted blunt trauma than the use as e.g. dressage horse [52]. This has a potential impact on the severity of tendon lesions and during the long term rehabilitation after 24 weeks.

Fiftyfive percent of the horses included in the current study had ultrasonographic signs of marginal/peripheral SDFT lesions which was considered as a high proportion compared to centrally located tendon lesions frequently diagnosed in race- and performance horses [4]. The etiology of peripheral tendon lesions has not been investigated separately. However, it has been assumed that these lesions typically occur in show jumpers, dressage horses [52] and cutting horses [53]. They may be attributed to degeneration or blunt trauma, e.g. self-kicking with the ipsilateral hind hoof, or to excessive strain on the lateral aspect of the affected limbs [53]. By contrast centrally located (“core”) lesions are the result of repetitive micro-injuries at high speed exercise and consecutive repair with insufficient tissue [3]. This lesion pattern was detected in only 3 horses in the current study population which predominantly consisted of warmblood horses used for jumping and dressage purposes.

This might explain why the effect of a single PRP injection on several parameters of tendon healing study was less profound in the current than in the surgical model used by Bosch et al. (2010) [14] that mimics central core-lesions after single macro-trauma in young mature horses, thus no effects of underlying ageing and/or degeneration. A recent systematic literature review revealed that generally more experimental, especially *in vitro* PRP studies yield positive results than clinical trials do, which proves that experimental settings only imperfectly replicate conditions of natural tendon repair [18]. Although the number of peripheral tendon lesions was similar in both groups of the current study, different lesion patterns potentially respond differently to intralesional injections. Fluid might more easily reflux to the subcutis after injection into marginally compared to centrally located lesions as the former are only surrounded by the peritendineum at their peripheral aspect. At the same time larger tendon lesions generally have a worse prognosis for functional repair [54] and CSA of peripheral lesions is small in most cases. It has been shown that peripheral tendon lesions in cutting horses required a mean convalescent period of 4 months before horses returned to full exercise [53] which is shorter than the usual rehabilitation period for other SDFT injuries [2].

In the current study all tendon lesions were injected with the same volume of PRP or saline to avoid an impact of a variable volume on the outcome. This was practiced inconsistently in previous studies including injections of naturally occurring, i.e. non-standardized tendon or ligament lesions with variable dimensions [16, 55]. A volume of 3 ml was chosen based on encouraging results of an equine study using the same volume in an experimental model of SDFT tendinopathy [14]. Volumes used in previous case series mostly focusing on suspensory ligament desmopathy varied between 0.5 and 12 ml [16, 17, 19, 55]. The volume chosen in the current study seemed to be appropriate as no resistance of the syringe plunger occurred during injection of the SDFTs being slightly under tension in a weightbearing position.

The volumes of PRP and saline were evenly distributed to three injection sites in the current study. A comparable approach was chosen in a human study comparing the effect of PRP and saline on chronic Achilles tendinopathy [20]. Saline led to similar improvements of the tendon structure as PRP which might indicate that also multiple injections with tiny volumes of sterile saline at one single time-point may trigger a healing response.

The number of horses with owner consent for a controlled clinical trial is generally limited and the current study was conducted in an equine referral clinic where horses are usually presented within several weeks after onset of clinical signs of tendinopathy. This led to the decision to include horses with a history of clinical signs of tendinopathy for up to 8 weeks. It had to be accepted that some tendons were most probably already in the late proliferative or even in the remodelling phase of tendon healing while most were in the late inflammatory and early proliferative phase. By contrast mechanically induced core lesions were treated as early as 1 week after creation of lesions, i.e. at the end of a well-defined acute inflammatory phase in the experiment by Bosch et al. (2011) [13]. There is evidence that timing of intralesional tendon treatment has a significant influence on outcome supported by a comparative experimental equine investigation showing that PRP treatment 7 days after SDFT lesion creation led to an earlier improvement of ultrasonographic parameters than treatment on day 14 [56]. It is known that growth factors from the α-granula of platelets such as PDGF and TGF-ß have a positive influence during the inflammatory phase of tendon healing: [57–59]. In an experimental model of rat collateral ligament injury the effectiveness of PDGF dropped markedly if it was administered more than 24 h after injury [60]. This is in accordance with results after treatment of chronic human Achilles tendinopathy which showed no beneficial effects of PRP versus placebo treatment with sterile saline [20]. At the same time PRP injections have been reported to be beneficial in chronic human tendon disorders refractory to other treatment modalities in several uncontrolled clinical studies [23, 61]. During the proliferation and remodelling phase of tendon healing Vascular Endothelial Growth Factor, which is a major component of PRP, might play a role as promotor of angiogenesis [58].

Horses in the current study received a single PRP treatment. This decision was based on the finding that growth factor and cytokine release from platelets continues from their mRNA reserves for at least 7 days after activation [62] and on a significant and long-lasting effect of a single injection approach in an experimental equine tendinopathy study [14]. Another reason was the intention to investigate a low dose of PRP as a basis for further research in equine naturally occurring tendinopathies [33], because to date no dose dependent studies are available with regard to treatment of equine tendinopathies. The limited long term effect in the current study may have been influenced by the fact that most growth factors contained in PRP are *per se* short lived which probably contributed to a low concentration over time in the clinical setting chosen. In a study using a rodent experimental model it was also shown that a single injection of heterologous PRP immediately after Achilles tendon transection only had time-limited effects on tendon healing during the observation period of up to 6 weeks [63]. Results of another experimental study in a rodent model of tendinosis suggested that a single intratendinous injection of PRP does not lead to a local and systemic increase in several cytokines and growth factors over the observation period of 25 days [64].

The adequate number of injections over time is also a point of debate in naturally occurring human tendinopathy: In a long term clinical trial in humans it was shown that a second early PRP injection does not rapidly improve clinical signs of tendinopathy in case of incomplete resolution after the first treatment [65]. However, it has been shown in a controlled human clinical trial that patients with chronic patellar tendinopathy receiving two injections of a leucocyte poor platelet concentrate (autologous conditioned plasma®) showed better improvement in outcomes when compared to a monoinjection [66]. Accordingly application of 3 consecutive PRP injections significantly improved clinical symptoms and function of human athletes with chronic patellar tendinopathy [22]. Summarily it cannot be excluded that more than one intralesional treatment might have enhanced the clinical effects observed in the current study.

Based on whole blood platelets and leucocytes were concentrated by a mean factor of 5.67 and 1.81, respectively in PRP in the current study. Compared to other PRP systems the bag system used in this study leads to an intermediate platelet and low leucocyte concentration [24]. By contrast platelets and leucocytes were enriched with factors 3.78 and 6, respectively, utilizing a different double centrifugation system in an equine surgical model of tendinopathy [14]. Among various other factors the different concentrations of platelets and leucocytes might have contributed to the differences in outcome between the latter and the current study.

Low platelet counts have been demonstrated to be inefficient in bone regeneration [67] and increasing platelet concentrations in a leucocyte reduced PRP preparation leads to delivery of more anabolic and less pro-inflammatory cytokines *in vitro* [28]. However, with increasing platelet concentrations the synthesis of collagen type I and III decreases suggesting a diminishing effect on tendon metabolism [28]. This observation corresponds to a dose dependent *in vitro* investigation showing that intermediate platelet concentrations, such as used in the current study, have stronger effects on tenocyte proliferation, migration and collagen production than highest concentrations [68].

While some beneficial effects such as anti-infectious properties [69], growth factor content [70] and promotion of chemotaxis, proliferation and differentiation of cells [71] have been attributed to leucocytes they also contribute to a greater acute inflammatory response [29, 72] and to the expression of catabolic factors, e.g. MMPs and IL-1, that may influence tendon healing negatively [27, 73, 74], so that a low leucocyte content seems to be preferable to enhance anabolic effects during tendon healing.

In summary PRP characteristics vary significantly depending on the technique and preparation kit used for its production [24, 75] which has a significant impact on clinical outcome and makes comparison between clinical trials difficult [61]. The optimal platelet to leucocyte ratio for treatment of tendinopathy is unknown to date and most probably dependent on the stage of tendon healing [76].

Clinical parameters did not differ between the PRPG and controls at any time point which corresponds to the results of an equine PRP study using an experimental model of tendinopathy [14]. However, clinical signs of inflammation such as the degree of lameness and scores for skin surface temperature decreased earlier in the PRPG than in the CG which may be attributed to anti-inflammatory and analgesic effects of PRP [40, 77, 78]. This correlates well to reduced pain scores in humans after PRP treatment of tendinopathy [79, 80] and to uncontrolled observations in two horses that returned to competition level performance as soon as 4,5 months after PRP treatment of SDFT lesions [17]. However, mean scores for sensitivity to palpation decreased earlier in controls than in PRP treated tendons despite a similar curve progression in both groups. This is potentially attributed to a high interindividual variation of score values due to the inclusion of different lesion patterns. Established semi-quantitative clinical score systems were used to monitor clinical parameters in the current study. Accuracy could have been increased by the use of computerized gait analysis and thermography [81].

With regard to B-mode ultrasonography total cross sectional area (T-CSA) decreased significantly between week 4 and 18 after PRP treatment while it did not change throughout the observation period in the CG. Sensitivity of CSA is potentially low to detect minor changes as shown by routine ultrasound assessment of racehorses [82]. However, a delayed decrease of T-CSA after the inflammatory phase of tendon healing can be interpreted as a therapeutic effect of the PRP injection.

In contrast to controls, scores for echogenicity were significantly higher which implies a low echogenicity of the tendon lesions at 4, 8 and 12 weeks after PRP treatment and at week 24, i.e. at the end of the observation period. Similarly scores for fiber alignment were significantly higher in the PRPG than in the CG at 4, 8 and 12 weeks after treatment suggesting a lower degree of continuity of fibrous structures. A potential explanation for the differences between groups is that TES and FAS as determined by B-mode were higher in the PRP group from the beginning of the experiment onwards, as comparison of the different time points reveals a tendency to a significant difference (*p* = 0.05−0.1) for most of the remaining time points. Consequently the intermittent significant difference between groups should be interpreted with caution. However, it cannot be excluded that PRP injection effectively induced a transient inflammatory response [29, 83] which might have contributed to a temporary decrease in echogenicity and in alignment of fibrous structures. This contrasts with results of an experimental equine study showing no differences at all for several B-mode parameters between SDFTs treated with a leucocyte poor single centrifugation plasma product and saline [84]. Although echogenicity and fiber alignment scoring using B-mode ultrasonograms are established tools in clinical settings, they have a limited sensitivity to adequately reflect tendon intergrity [35]. Additionally lesion types included in the current study were heterogenous and the number of cases per group was relatively low. These factors made B-mode ultrasonography not the ideal modality to comparatively monitor especially subtle changes during the process of tendon repair in the current study. It could be proved that echogenicity and fibre alignment scores based on B-mode ultrasonograms do not correspond to the degree of echo continuity as determined by UTC implying a higher sensitivity of the latter technique [35, 37].

During ultrasound tissue characterization of PRPG tendons there was a significant drop in the proportion of non-structure related echo-type type IV representing a mainly amorphous matrix and/or free fluid between day 0 and week 12 while proportions for this echo type remained on the same level in the CG throughout 24 weeks. This phenomenon was previously observed in an experimental equine study after a single PRP injection and may be attributed to acute anti-inflammatory effects exerted by PRP [40, 77, 78].

The decreasing proportions of non-structure related echo types III and IV between day 0 and week 12 after PRP treatment in the current study rather resembles the course between week 4 and 12 after surgical induction of SDFT lesions without treatment [39] and the progression between week 5 and 18 after PRP treatment in another experimental equine study [40] and might therefore be an indicator for the chronicity of some tendon lesions included in the current study population. Hence, the delayed treatment up to 8 weeks after the onset of clinical signs might have contributed to a right-shift of the healing pattern as determined by UTC compared to experimental tendon lesions. However, more frequent UTC examinations between day 0 and week 12 would have been necessary to further prove this. This could not be realized due financial constraints and organisatory reasons as all horses were client owned and horses have to be sedated for 30–60 min during UTC scanning.

Between week 12 and week 24 combined echo-types I and II (fascicular structures) increased significantly within the PRPG. This resulted in a significantly higher percentage of echo type II being representative for the organization of a fibrillary matrix into fascicles that are still discontinous and not yet aligned, thus still remodelling after 24 weeks in PRP treated compared to control tendons. An echo-type II ratio described for normal tendon tissue, i.e. approximately 0.18 [39], was almost reached. In combination with a significant decreases of summarized echo-types III and IV between week 12 and week 24 this indicates an advanced organization of fibrillar and amorphous matrix into fascicles that are less continuous, more swollen and not aligned yet. The latter interpretation is supported by a lack of significant differences between groups with regard to echo-type I proportions representing intact and fully aligned fascicles (secondary tendon bundles).

In contrast to the current study PRP treated SDFTs had a significantly lower percentage of echo type II than controls in a surgical tendon model at the end of the observation period of 24 weeks [40]. This indicates that in the Bosch et al. study, using a single macro-trauma, the PRP-treated tendons passed already the remodelling stage while during the experiment described in this manuscript the fascicles were still not aligned yet and still requiring remodelling. In general the higher proportion of echo-type II in the PRPG is an indicator of an active process of remodelling 24 weeks after treatment [40].

The proportion of echo type III being representative for mainly fibrillar matrix continued to decrease significantly between week 12 and 24 only in the PRPG. Comparison of the proportion of this echo type to others in the PRPG suggests that the decrease in echo type III is rather in favour of echo type I than in favour of echo type II between week 12 and 24 as could be expected from a previous study [39]. Main reason for this observation is that in our study there are only limited time-points for UTC scanning, missing some detailed information on stages of repair. Anyhow, this finding suggests a transformation from fibrillar components into intact and aligned tendon bundles during the remodelling phase after PRP treatment of naturally occurring tendinopathies.

All horses included in the current study underwent the same controlled exercise regimen as it is known that controlled exercise has a therapeutic effect on tendon healing [85]. The duration of the controlled exercise prescribed for the included horses started on the basis of the estimated duration of tendinopathy (<4 weeks or 5–8 weeks) to adequately address the potentially therapeutic effect of gradually increasing controlled exercise on the process of tendon healing in each individual. To avoid inadequate loading of the tendons immediately after treatment all horses were given 3 days box rest and the first actual exercise level was restricted to 10 min walk.

Similarly all horses were shod with a closed horse shoe with a straight bar because this type of shoeing has been described as adequate during rehabilitation in cases of SDFT tendinopathy [49]. Despite potential alternative approaches to decrease strain in the SDFT via orthopaedic shoeing [86] authors intended to assure that rehabilitation conditions including shoeing were as similar as possible for all horses which seemed to be achievable most consistently with the type of shoeing chosen.

Recurrence rate is one of the most critical parameters of long-term functionality after tendon injury [31]. Only 1 out of 10 horses in either group developed recurrence of tendinopathy in the treated limb within 24 months after treatment. This is a lower recurrence rate than that previously reported after conventional intralesional treatment of SDF tendinopathy in different types of performance horses [2]. However, it has to be considered that several previous equine tendon studies mainly included racehorses typically developing centrally located core lesions [3, 31] which probably have a different etiology than most of the tendon lesions in the current study [53]. Due to the relatively low number of horses included and different types of use, recurrence rate in the current study should be interpreted cautiously. Biomechanical testing using tendon explants is the gold standard to determine elasticity and strength of tendon tissue. Due to the inclusion of client owned horses it could not be performed in the current study.

The performance level which is another important indicator of functionality did not differ statistically between groups at 12 and 24 months after treatment. However, the current study revealed that 8 versus 5 horses reached their previous level of performance 12 months after PRP and saline treatment respectively. After another 12 months only 6 of the PRP treated and 5 control horses performed at their previous level. This finding reflects the results of a clinical study including racehorses with midbody suspensory ligament demopathies [16]. Horses treated with PRP had fewer starts than control horses delayed only during the third year after return to racing.

## Conclusions

This controlled clinical trial shows that a single intralesional injection of PRP up to 8 weeks after onset of clinical signs of tendinopathy contributes to an earlier reduction of lameness compared to a single intralesional treatment with saline. Compared to controls fibrillar matrix in PRP treated tendons is getting organized into fascicles that are not aligned yet after six months which is a sign of advanced tendon repair while remodelling continues. PRP treatment has the potential to increase the number of horses reaching their previous or a higher level of performance after 12 months which is not sustained after 24 months. Earlier treatment of tendinopathy with PRP should be considered to enhance these effects. Future controlled clinical studies should include higher numbers of horses.

## Abbreviations

ACD-A: Acid citrate dextrose-A
CG: Control group
DICOM: Digital imaging and communications in medicine
EDTA: Ethylendiamintetraacetate
G: Gauge
IV: Intravenously
MIZ: Maximal injury zone
PDGF-BB: Platelet derived growth factor-BB
PPP: Platelet poor plasma
PRP: Platelet-rich plasma
PRPG: Platelet-rich plasma-treated group
SDFT: Superficial digital flexor tendon
SEM: Standard error of the mean
T-CSA: Total cross-sectional area
TES: Total echo score
T-FAS: Total fibre alignment score
TGC: Time gain compensation
TGF-ß: Transforming growth factor-ß
US: Ultrasound
UTC: Ultrasound tissue characterization.
